# Child diarrhoea and nutritional status in rural Rwanda: a cross‐sectional study to explore contributing environmental and demographic factors

**DOI:** 10.1111/tmi.12725

**Published:** 2016-06-16

**Authors:** Sheela S. Sinharoy, Wolf‐Peter Schmidt, Kris Cox, Zachary Clemence, Leodomir Mfura, Ronald Wendt, Sophie Boisson, Erin Crossett, Karen A. Grépin, William Jack, Jeanine Condo, James Habyarimana, Thomas Clasen

**Affiliations:** ^1^ Nutrition and Health Sciences Program Laney Graduate School Emory University Atlanta GA USA; ^2^ Faculty of Infectious and Tropical Diseases London School of Hygiene and Tropical Medicine London UK; ^3^ Innovations for Poverty Action New Haven CT USA; ^4^ McCourt School of Public Policy Georgetown University Washington DC USA; ^5^ Robert F. Wagner Graduate School of Public Service New York University New York NY USA; ^6^ Rwanda Biomedical Center Kigali Rwanda; ^7^ Rollins School of Public Health Emory University Atlanta GA USA

**Keywords:** drinking water, sanitation, diarrhoea, nutrition, stunting, eau potable, sanitaire, diarrhée, nutrition, retard de croissance

## Abstract

**Objective:**

To explore associations of environmental and demographic factors with diarrhoea and nutritional status among children in Rusizi district, Rwanda.

**Methods:**

We obtained cross‐sectional data from 8847 households in May–August 2013 from a baseline survey conducted for an evaluation of an integrated health intervention. We collected data on diarrhoea, water quality, and environmental and demographic factors from households with children <5, and anthropometry from children <2. We conducted log‐binomial regression using diarrhoea, stunting and wasting as dependent variables.

**Results:**

Among children <5, 8.7% reported diarrhoea in the previous 7 days. Among children <2, stunting prevalence was 34.9% and wasting prevalence was 2.1%. Drinking water treatment (any method) was inversely associated with caregiver‐reported diarrhoea in the previous 7 days (PR = 0.79, 95% CI: 0.68–0.91). Improved source of drinking water (PR = 0.80, 95% CI: 0.73–0.87), appropriate treatment of drinking water (PR = 0.88, 95% CI: 0.80–0.96), improved sanitation facility (PR = 0.90, 95% CI: 0.82–0.97), and complete structure (having walls, floor and roof) of the sanitation facility (PR = 0.65, 95% CI: 0.50–0.84) were inversely associated with stunting. None of the exposure variables were associated with wasting. A microbiological indicator of water quality was not associated with diarrhoea or stunting.

**Conclusions:**

Our findings suggest that in Rusizi district, appropriate treatment of drinking water may be an important factor in diarrhoea in children <5, while improved source and appropriate treatment of drinking water as well as improved type and structure of sanitation facility may be important for linear growth in children <2. We did not detect an association with water quality.

## Abstract (fr)

**Objectif:**

Explorer les associations entre les facteurs environnementaux et démographiques avec la diarrhée et l’état nutritionnel chez les enfants dans le district de Rusizi, au Rwanda.

**Méthodes:**

Nous avons obtenu des données transversales de 8.847 ménages de mai à août 2013 d'une enquête de référence réalisée pour l’évaluation d'une intervention de santé intégrée. Nous avons recueilli les données sur la diarrhée, la qualité de l'eau et les facteurs environnementaux et démographiques des ménages avec des enfants <5ans, et l'anthropométrie des enfants <2 ans. Nous avons effectué une régression log‐binomiale en utilisant la diarrhée, le retard de croissance et l’émaciation comme variables dépendantes.

**Résultats:**

Chez les enfants <5 ans, 8,7% ont rapporté la diarrhée au cours des 7 jours précédents. Chez les enfants <2 ans, la prévalence du retard de croissance était de 34,9% et celle de l’émaciation de 2,1%. Le traitement de l'eau de boisson (toute méthode) était inversement associé à la diarrhée rapportée par les soignants au cours des sept jours précédents (PR = 0,79; IC95%: 0,68 à 0,91). L'amélioration de la source d'eau de boisson (PR = 0,80; IC95%: 0,73 à 0,87), un traitement approprié de l'eau de boisson (PR = 0,88; IC95%: 0,80 à 0,96), l'amélioration des installations sanitaires (PR = 0,90; IC95%: 0,82‐0,97) et une structure complète (présence de murs, de plancher et de toit) de l'installation sanitaire (PR = 0,65; IC95%: 0,50 à 0,84) étaient inversement associés à un retard de croissance. Aucune des variables d'exposition n'a été associée à l’émaciation. Un indicateur microbiologique de la qualité de l'eau n'a pas été associé à la diarrhée ou au retard de croissance.

**Conclusions:**

Nos résultats suggèrent que dans le district de Rusizi, un traitement approprié de l'eau de boisson peut être un facteur important dans la diarrhée chez les enfants <5 ans, tandis que l'amélioration de la source et un traitement approprié de l'eau de boisson ainsi que l'amélioration du type et de la structure des installations sanitaires peuvent être importants pour une croissance linéaire chez les enfants <2 ans. Nous n'avons pas détecté une association avec la qualité de l'eau.

## Abstract (es)

**Objetivo:**

Explorar las asociaciones entre factores ambientales y demográficos con la diarrea y el estatus nutricional de niños del distrito de Rusizi, Ruanda.

**Métodos:**

Hemos obtenido datos croseccionales de 8,847 hogares entre Mayo y Agosto del 2013 de una encuesta basal realizada por la evaluación de una intervención integral en salud. Hemos recogido datos sobre diarrea, calidad del agua, factores ambientales y demográficos de hogares con niños <5 años, y antropometría de niños <2 años. Hemos realizado una regresión logística binaria utilizando diarrea, la atrofia y el marasmo como variables dependientes.

**Resultados:**

De los niños <5 años, un 8.7% reportó diarrea en los últimos 7 días. Entre los niños <2 años, la prevalencia de atrofia era del 34.9% y la prevalencia de marasmo del 2.1%. El tratamiento del agua para consumo (cualquier método) estaba inversamente asociada con la diarrea reportada por el cuidador en los siete días previos (PR=0.79, IC 95%: 0.68‐0.91). Una fuente mejorada del agua para consumo (PR=0.80, IC 95%:0.73‐0.87), el tratamiento apropiado del agua para consumo (PR=0.88, IC 95%:0.80‐0.96), mejoras en las instalaciones sanitarias (PR=0.90, IC 95%:0.82‐0.97) y una estructura completa (tener paredes, suelo, y techo) de las instalaciones sanitarias (PR=0.65, IC 95%:0.50‐0.84) estaban inversamente asociados con atrofia. Ninguna de las variables de exposición estaba asociada con el marasmo. Un indicador de la calidad del agua no estaba asociado con la diarrea o la atrofia.

**Conclusiones:**

Nuestros hallazgos sugieren que en el distrito de Rusizi, el tratamiento apropiado del agua para consumo podría ser un factor importante en la diarrea de niños <5 años, mientras que las mejoras en las fuentes y un tratamiento adecuado del agua para consumo, al mismo tiempo que el tipo y estructura de las instalaciones sanitarias podrían ser importantes para el crecimiento linear en niños <2 años. No detectamos una asociación con la calidad del agua.

## Introduction

Globally, diarrhoea and undernutrition together contribute to a large proportion of deaths among children under 5 years old. Diarrhoea is second only to pneumonia as the leading cause of post‐neonatal death, accounting for an estimated 700 000 deaths annually in this age group 1. Undernutrition is the largest single underlying cause of death among children <5, playing a role in nearly 3 million deaths per year 2. Diarrhoea contributes to undernutrition through multiple pathways, including reduced energy intake, nutrient loss and malabsorption 3, 4. Undernutrition in turn reduces the body's defences against infection, potentially creating a vicious cycle 4, 5.

Water, sanitation and hygiene (WASH) are linked to diarrhoea and nutrition through multiple pathways. Faecal exposure through contaminated water, unimproved sanitation and poor hygiene can lead to diarrhoea and subclinical infection, including environmental enteric dysfunction, both of which are negatively associated with child growth 6, 7. Poor WASH practices also increase the risk of intestinal parasitic infection, which can impact child nutrition and growth through anaemia and appetite suppression 8.

Measures of child undernutrition include stunting (length‐for‐age *z*‐score <−2), which represents long‐term nutritional status, and wasting (weight‐for‐length *z*‐score <−2), which represents acute nutritional status. Stunting is a serious problem with long‐term negative sequelae for cognitive development, educational achievement and adult productivity and income 9, 10, 11, 12. Both stunting and wasting can increase a child's risk of mortality, though wasting has a stronger association with mortality 2, 13.

In Rwanda, the 2014–2015 Demographic and Health Survey (DHS) Key Indicators report estimates that 12% of children <5 nationally had caregiver‐reported diarrhoea in the previous 2 weeks, 38% of children were stunted, and 2% of children were wasted 14. This represents a slight, though likely not meaningful, decline in diarrhoea prevalence, from a prevalence of 13% in 2010 15. For stunting, this represents a steady decrease from a prevalence of 51% in 2005 and 44% in 2010, bringing the prevalence in line with the regional average of 39% for Eastern and Southern Africa 15, 16, 17. For wasting, the prevalence also represents a decrease from 5% in 2005 15 and is substantially lower than the regional prevalence of 6.9% for eastern and southern Africa 17.

Despite the high prevalence of diarrhoea and stunting, there are few published studies on their determinants among children in Rwanda. We aimed to redress this research gap by examining associations of these outcomes with environmental and demographic factors.

## Methods

### Data sources

The data for this study come from a baseline survey conducted as part of a cluster‐randomised controlled trial to assess the impact of the Community‐Based Environmental Health Promotion Programme (CBEHPP). CBEHPP is a program of the Rwandan Ministry of Health that aims to achieve zero open defecation, at least 80% hygienic latrine coverage, and improvements in related health behaviours such as household water treatment and hand‐washing with soap. The trial covers 150 villages that were randomly selected from the 598 villages in Rusizi district. Rusizi was chosen because it had no existing donor support for environmental health and had a higher burden of sanitation‐ and hygiene‐related diseases, including reported diarrhoea, than other candidate districts. A baseline survey was conducted from May to August 2013. All 150 villages were visited, and all households with children under age five were targeted for inclusion in the study. A total of 8847 households (with 13 278 children under age five) consented to participate in the study and were enrolled.

Data collection methods included a structured survey tool as well as observation of household latrines and handwashing stations. Latrines were observed for structural qualities (i.e. existence of a floor, roof and walls) and cleanliness (i.e. absence of visible faeces on the floor and/or walls). Handwashing stations were checked for availability of water and soap. Prevalence of diarrhoea among children <5 was measured by asking the child's mother or other caregiver whether the child had suffered from diarrhoea within the past 7 days. Diarrhoea was defined using the WHO definition of three or more loose stools (that can take the shape of a container) within a 24‐h period 19.

Anthropometric data were collected for all children under age two in the participating households (*N* = 5062). Children <2 were targeted because of the critical importance of the first 24 months of life for linear growth 20, 21. Where possible, for children <2, age was verified using birth certificates and immunisation cards. Weight was measured using a SECA 385 scale, with 20 g increment for weight below 20 kg and 50 g increment for weight between 20–50 kg. Recumbent length was measured with SECA 417 boards with 1 mm increments.

Water samples were collected from approximately 10% of participating households (*N* = 900), randomly selected in each study village. Trained field staff collected 125‐ml samples from households’ drinking water containers in sterile Whirl‐Pak^™^ Bags (Nasco International, Fort Atikinson, WI, USA). Samples were placed on ice and processed within 4 h of collection to assess levels of thermotolerant (faecal) coliforms (TTC), a well‐established WHO indicator organism for faecal contamination 18. Microbiological assessment was performed using a membrane filtration method with membrane lauryl sulphate medium using a DelAgua field incubator in accordance with the Standard Methods 22. Of this sample, 1345 children under five had data on both water quality and diarrhoea, and 488 children under two had data on both water quality and anthropometry.

### Variables

The primary outcome was diarrhoea among children <5 in the past 7 days. Secondary outcomes were stunting and wasting. To determine whether children were stunted or wasted, we calculated length‐for‐age z‐scores (LAZ) and weight‐for‐length *z*‐scores (WLZ) using WHO reference standards. Children with LAZ < −2 were considered stunted, and those with LAZ ≥ −2 were considered not stunted. In all analyses, LAZ scores <−6.00 or >6.00 were considered outliers and excluded from the study (*N* = 86). Similarly, children with WLZ < −2 were considered wasted, and those with WLZ ≥ −2 were considered not wasted. In all analyses, WLZ scores <−6.00 or >6.00 were considered outliers and excluded from the study (*N* = 41).

The independent variables selected *a priori* for this analysis focused on environmental factors that can affect faecal exposure, such as observed presence of a handwashing station with soap and water, source of drinking water, treatment of drinking water, type of sanitation facility, whether sanitation facility is shared, observed cleanliness of sanitation facility, structure of sanitation facility and method of disposal of child faeces. Caregiver‐reported diarrhoea in the previous 7 days was used as an independent variable in analyses of anthropometric outcomes. We created an asset index by applying principal component analysis to measures of asset ownership, including ownership of household goods, livestock and land, as well as characteristics of the housing structure 23. The asset index was used as a proxy for socio‐economic status. Other covariates included child age in months, sex of the child and years of maternal schooling.

Colony‐forming units (CFU) of TTC per 100 ml water were used as a continuous independent variable in separate analyses. Values with CFUs too numerous to count were replaced with 300 CFU per ml. Data for this variable were non‐normally distributed, hence we calculated Williams means by adding one to all values then calculating the geometric mean and subtracting one. This variable was categorised as very high, high, moderate, poor and very poor quality water based on cut‐offs of <1, 1–10, 11–100, 101–1000 and >1000 CFU per 100 ml 24.

### Statistical analyses

We calculated descriptive statistics, then tested univariable relationships between each outcome and exposure variables of interest. Based on the descriptive statistics, we dropped presence of an observed handwashing station with soap and water as an exposure variable due to the small number of children who had both an observed handwashing station in their household and any of the outcomes (*N* ≤ 10). For each outcome variable (diarrhoea, stunting and wasting), we used log‐binomial regression with a log link function and generalised estimating equations (GEE), then exponentiated the coefficients to obtain prevalence ratios (PRs). For each exposure variable, we then created separate models adjusting for confounders and calculating adjusted prevalence ratios. We identified confounders based on univariable analyses, defining a confounder as any variable that was associated with both the outcome and the exposure variables and not on the causal path between the exposure and the outcome 25. For wasting, some exposure variables had no recognised confounders, so adjusted prevalence ratios were not calculated. In all models, we calculated robust standard errors and used the household ID as a group variable to account for clustering at the village and household level.

Due to the smaller number of children under five (*N* = 1345) and under two (*N* = 488) with both quantitative household water quality measurements and outcome data, we carried out separate analyses of associations with water quality. We included water quality as a categorical exposure variable to examine associations by risk category. Due to the small number of households (*N* = 3) with water in the very poor quality category, we combined this category with the poor quality category. For diarrhoea and stunting, we calculated crude and adjusted prevalence ratios for the associations with water quality. Due to the small number of children who were categorised as wasted and who had data for household water quality (*N* = 7), we did not examine associations of water quality and wasting.

Statistical analyses were conducted using STATA version 13.1 (College Station, TX).

### Ethics

This secondary analysis was conducted as part of larger cluster randomised trial, for which the protocol was reviewed and approved by the Rwanda National Ethics Committee and the Institutional Review Board of Innovations for Poverty Action. The trial is registered with ClinicalTrials.org (NCT01836731). The analysis presented here used de‐identified secondary data and human subjects review was not required.

## Results

### Characteristics of study households, mothers and children

Among children <5, diarrhoea prevalence was 8.7% (Table 1). Among children <2, diarrhoea prevalence was 13.5%, stunting prevalence was 34.9% and wasting prevalence was 2.1%. After accounting for difference in diarrhoea reporting between surveys, these figures correspond to estimates from the DHS. In the sample of households for which water quality data were available, 27.8% of children <2 (*N* = 525) and 28.2% of children <5 (*N* = 1369) lived in households where the water was of very high quality (<1 CFU per 100 ml). Only 0.4% of children <2 and 0.2% of children <5 lived in households where the water was of very poor quality (>1000 CFU per 100 ml).

**Table 1:** Descriptive statistics of children age 0–23 and 0–59 months in CBEHPP study area, Rusizi District

Background characteristic	Children <24 months		Children <60 months	
	*N*	Percent	*N*	Percent
Diarrhoea in previous 7 days	5056		12 888	
Yes		13.5		8.7
HAZ [mean (SD)]	4885	−1.51 (1.41)	–	
Stunted		34.9		–
WHZ [mean (SD)]	4909	0.27 (1.14)		
Wasted		2.1		–
*E. coli* CFU/100 ml	525		1369	
1–10		20.2		20.8
11–100		24.8		24.6
101–1000		26.9		26.2
>1000		0.4		0.2
Age	5212		13 254	
0–5 months		24.3		9.5
6–11 months		23.7		9.3
12–23 months		52.1		20.5
21–59 months				60.7
Sex	5211		13 253	
Female		50.6		49.9
Maternal schooling	5029		12 569	
Years [mean (SD)]		4.3 (3.0)		4.1 (3.0)
Source of drinking water	5212		13 254	
Improved		74.6		75.3
Drinking water treatment	5212		13 254	
Appropriate		32.4		31.1
Observed handwashing station with soap and water	5212		13254	
Yes		1.1		1.1
Practice open defecation	5212		13 254	
Yes		2.1		2.0
Type of sanitation facility	5212		13 254	
Improved		67.5		66.7
Shared sanitation facility	5212		13 254	
Yes		18.2		17.3
Sanitation facility cleanliness	5138		13 072	
No visible faeces on walls or floor		19.8		20.7
Sanitation facility structure	5138		13 072	
Has floor + walls + roof		5.9		5.6
Disposal of child faeces	5138		13 254	
Sanitary		85.0		89.9
Wealth quintile	5107		12 997	
Second		20.5		20.7
Third		20.2		20.9
Fourth		19.8		19.4
Fifth		19.1		18.9

The majority of children lived in households with improved drinking water sources, improved sanitation facilities and sanitary disposal practices for child faeces. Nearly one‐third of children lived in households that reported using appropriate water treatment methods. Among households with an improved source of drinking water, 32.4% reported using an appropriate method of water treatment, *vs*. 29.5% among those with an unimproved water source. This difference was statistically significant (*P* = 0.010; data not shown). Fewer than 20% of children lived in household where a sanitation facility was shared with at least one other household. Handwashing stations with soap and water were rare and were observed in only 1% of households.

### Associations between diarrhoea, stunting and wasting and independent variables

Table 2 shows the results of crude and adjusted log‐binomial regression models with diarrhoea, stunting and wasting as outcomes. Only one variable, adequate treatment of drinking water, was associated with caregiver‐reported diarrhoea among children <5 in adjusted models (PR = 0.79, 95% CI: 0.68–0.91). This PR indicates children whose household reported using adequate methods of treating their drinking water were 21% less likely to have had diarrhoea in the previous 7 days than children whose households reported inadequate or no methods of treating their drinking water. Type of sanitation facility was less strongly associated with diarrhoea (PR = 0.89, 95% CI: 0.78–1.02). The other exposure variables (source of drinking water, practice of open defecation, shared sanitation facility, cleanliness and structure of sanitation facility, and disposal of child faeces) were not associated with diarrhoea. As with children <5, results indicate that adequate drinking water treatment is the only important predictor of diarrhoea in children <2 (data not shown).

**Table 2:** Log‐binomial regression models of prevalence ratios associated with diarrhoea among children 0–59 months, and stunting and wasting among children 0–23 months, in the CBEHPP study area, Rusizi District

	Univariable prevalence ratio (95% confidence limit)	Multivariable prevalence ratio (95% confidence limit)	*P* value^a^	*N*
Exposure variables of interest				
Outcome: diarrhoea in previous 7 days among children under 5				
Source of drinking water^b^	0.90 (0.79, 1.02)	0.93 (0.81, 1.06)	0.266	12 359
Drinking water treatment^c^	0.75 (0.65, 0.86)	0.78 (0.68, 0.90)	0.001	12 359
Practice open defecation^d^	1.40 (0.99, 1.98)	1.31 (0.93, 1.85)	0.119	12 359
Type of sanitation facility^d^	0.82 (0.73, 0.93)	0.89 (0.78, 1.02)	0.090	12 359
Shared sanitation facility^c^	1.15 (0.99, 1.33)	1.10 (0.94, 1.27)	0.226	12 359
Cleanliness of sanitation facility^b^	1.00 (0.87, 1.16)	0.96 (0.83, 1.11)	0.596	12 195
Structure of sanitation fadlity^b^	0.75 (0.56, 1.00)	0.85 (0.62, 1.16)	0.291	12 195
Disposal of child feces^c^	0.91 (0.75, 1.10)	1.13 (0.93, 1.38)	0.213	12 359
Outcome: stunting among children under 2				
Diarrhoea in previous 7 days^c^	1.13 (1.01, 1.26)	1.07 (0.96, 1.19)	0.204	4619
Source of drinking water^b^	0.79 (0.73, 0.86)	0.80 (0.73, 0.87)	<0.001	4650
Drinking water treatment^e^	0.80 (0.73, 0.88)	0.87 (0.80, 0.96)	0.005	4624
Practice open defecation^d^	1.58 (1.31, 190)	1.44 (1.21, 1.71)	<0.001	4624
Type of sanitation facility^d^	0.79 (0.73, 0.85)	0.90 (0.82, 0.97)	0.010	4624
Shared sanitation facility^c^	1.03 (0.93, 1.14)	1.01 (0.92, 1.12)	0.784	4624
Cleanliness of sanitation facility^b^	0.97 (0.88, 1.07)	0.94 (0.85, 1.04)	0.254	4563
Structure of sanitation facility^b^	0.59 (0.46, 0.74)	0.65 (0.50, 0.84)	0.001	4563
Disposal of child feces^c^	1.53 (1.33, 1.75)	1.04 (0.91, 1.19)	0.547	4624
Outcome: wasting among children under 2				
Diarrhoea in previous 7 days^f^	1.29 (0.77, 2.16)	1.34 (0.80, 2.23)	0.270	4723
Source of drinking water	0.96 (0.61, 1.49)	–	0.839	4870
Drinking water treatment	1.10 (0.73, 1.65)	–	0.660	4870
Practice open defecation	2.04 (0.74, 5.62)	–	0.166	4870
Type of sanitation facility^d^	1.09 (0.72, 1.67)	–	0.681	4870
Shared sanitation facility^c^	1.10 (0.68, 1.79)	1.11 (0.68, 1.81)	0.666	4870
Cleanliness of sanitation facility^f^	1.18 (0.74, 1.89)	1.14 (0.71, 1.82)	0.588	4802
Structure of sanitation facility	1.54 (0.78, 3.03)	–	0.212	4802
Disposal of child feces^f^	1.35 (0.74, 245)	1.59 (0.86, 294)	0.137	4870

a For the multivariable anaysis.

b Adjusted for maternal schooling and household wealth.

c Adjusted for child age in months, maternal schooling, and household wealth.

d Adjusted for source of drinking water, maternal schooling, and household wealth.

e Adjusted for child sex, maternal schooling, and household wealth.

f Adjusted for child age in months.

Among children <2, the prevalence of stunting was lower among children whose households reported having an improved source of drinking water (PR = 0.80, 95% CI: 0.73–0.87), treating their drinking water using adequate methods (PR = 0.88, 95% CI: 0.80–0.96), having an improved sanitation facility (PR = 0.90, 95% CI: 0.82–0.97), and where the sanitation facility was observed to be structurally complete (PR = 0.65, 95% CI: 0.50–0.84), and was higher among children whose households practice open defecation (PR = 1.44, 95% CI: 1.21–1.71). Caregiver‐reported diarrhoea in the previous 7 days, shared sanitation facility, cleanliness of the sanitation facility and method of disposal of child faeces were not associated with stunting. Wasting was not associated with any of the exposure variables. The results for wasting generally had substantially wider confidence intervals than the results for diarrhoea and stunting, indicating less precision in the estimates.

Table 3 shows results from log‐binomial regression models using diarrhoea and stunting as outcomes and water quality as the exposure variable. Among those households with data for water quality, a total of 1283 children <5 and 471 children <2 had data for diarrhoea and stunting, respectively. Of those children, 8.7% (*N* = 111) children <5 had diarrhoea and 35.2% (*N* = 166) children <2 were stunted. Water quality was not significantly associated with any of the outcomes in crude or adjusted models in this population.

**Table 3:** Log‐binomial regression models of prevalence ratios associated with diarrhoea among children 0–59 months, and stunting and wasting among children 0–23 months, in the CBEHPP study area, Rusizi District

	Univariable prevalence ratio (95% confidence limit)	Multivariate prevalence ratio (95% confidence limit)	*P* value^a^	*N*
Outcome: diarrhoea in previous 7 days among children under 5				
Water quality (CFU/100 ml)				1283
Reference: CFU = 0				362
1–10	0.80 (0.47, 1.35)	0.80 (0.47, 1.35)	0.395	267
11–100	0.80 (0.48, 1.36)	0.80 (0.47, 1.35)	0.405	310
>101	0.86 (0.53, 1.40)	0.77 (0.46, 1.29)	0.317	344
Outcome: stunting among children under 2				
Water quality (CPU/100 ml)				471
Reference: CFU = 0				130
1–10	1.27 (0.88, 1.83)	1.23 (0.86, 1.76)	0.263	95
11–100	1.32 (0.93, 1.86)	1.21 (0.85, 1.72)	0.296	111
>101	1.12 (0.78, 1.60)	1.00 (0.70, 1.45)	0.987	133

Analysis is adjusted for maternal schooling and household wealth.

a For the multivariable analysis.

## Discussion

We describe associations of health, sanitation, environmental and demographic factors with diarrhoea and nutritional status outcomes among children in Rusizi District, Rwanda. We found that adequate treatment of drinking water was inversely associated with caregiver‐reported diarrhoea among children under five. We found that improved source and adequate treatment of drinking water as well as improved type and structure of sanitation facility were inversely related to stunting, while open defecation was positively related to stunting, among children under two. None of the exposure variables were related to wasting. Quantitative measurements of water quality were not associated with either diarrhoea or stunting.

The lack of a clear dose−response relationship between diarrhoea and water quality is not unusual in the literature. A systematic review by Gundry *et al*. 26 of associations between diarrhoea among pre‐school children and microbiological indicators of water quality produced a pooled odds ratio of 1.12 (CI: 0.85–1.48). A subsequent meta‐analysis of water quality and diarrhoea by Gruber *et al*. 27 which included studies of adults and children, resulted in a relative risk of 1.26 (95% CI: 0.98–1.63). A more recent study from Bangladesh found a positive association with diarrhoea when the *Escherichia coli* concentration in drinking water was 100–999/100 ml but not when the concentration was ≥1000 28. It may be that faecal agents in drinking water are not diarrhoeagenic to household members who have been previously exposed to them, or other routes of exposure may be more dominant than the waterborne route 29.

Despite a lack of associations with water quality, reported adequate treatment of drinking water was inversely associated with both diarrhoea and stunting. The relationship with stunting is biologically plausible over the long term if consistent and regular water treatment reduced faecal pathogens that cause diarrhoea or environmental enteropathy, or if treatment reduced transmission of parasites. The inverse associations of source of drinking water, sanitation facility, and structure of sanitation facility, and the positive association of open defecation with stunting may also represent mechanisms through which transmission of faecal pathogens and parasites may be reduced, thereby benefiting children's nutrition. The lack of an association between cleanliness of sanitation facility and stunting may be due to the variable's definition (absence of observed faeces on the floor and/or walls of the sanitation facility) being too broad; finer measurements of cleanliness may be needed.

Other studies have found mixed results when examining WASH and child growth 30. Water quality was associated with linear growth in a study in India 31, and water source, water storage and sanitation were associated with child height at 2 years in a study in Peru 32. In contrast, source of drinking water was not associated with child growth in a study in Bangladesh 33. Associations between sanitation and linear growth have been found in some studies 33, 34, 35, 36 but not in others 37, 38. Similar evidence is lacking for Rwanda. The results of our study suggest that for young children in Rusizi district, both water and sanitation may be important factors in children's linear growth.

The lack of an association of wasting with diarrhoea was surprising, given the global evidence that diarrhoea is associated with weight loss in the short term 39, 40. The PR of 1.34 suggested a positive association, as would be expected from the literature, but the confidence interval (CI: 0.80–2.23) was wide and suggested a lack of precision. The very low prevalence of wasting, while desirable from a public health perspective, likely contributed to the imprecise estimates in analyses using this variable as an outcome.

This cross‐sectional study provided a baseline for CBEHPP, which targeted several of the factors associated with stunting, including improved sanitation facilities and associated health behaviours. It is hypothesized that improvements in health behaviours and sanitation infrastructure will be associated with a reduction in diarrhoea prevalence and improvements in mean LAZ among children in the intervention areas. The project's impact on child diarrhoea and nutritional status will be evaluated after the program has ended.

The results of this study suggest that programs focusing on improved water and sanitation infrastructure may hold promise for improving children's linear growth in this population. This is in line with other studies that have similarly hypothesised that focusing on water and sanitation may be beneficial for children's nutritional status 41, 42.

### Limitations

A key limitation of this study is the cross‐sectional design. A stronger design for examining these outcomes would have been a longitudinal study with weekly data collection on morbidity, which would have allowed us to relate morbidity during specific intervals to growth in length in these same intervals. In particular, diarrhoea and stunting are considered to have a dose–response relationship, in which repeated or persistent episodes of diarrhoea are associated with an increased risk of stunting at 24 months of age 43. A lack of regular, frequent data on these outcomes for each child can potentially lead to regression dilution bias 44. Given that our study examined diarrhoea prevalence only in one‐seven‐day period, it is not possible to know whether an association between diarrhoea and stunting exists over the long term in this population.

Other limitations of this work are the lack of data on sub‐clinical outcomes and potential confounders. We did not measure biomarkers of environmental enteropathy or children's micronutrient status, nor do we have data on children's dietary intake or household food security. We were also unable to analyse associations with handwashing due to the low prevalence of observed handwashing stations in study households. Finally, our study used caregiver report or self‐report for variables such as diarrhoea, water treatment and method of disposal of child faeces. These may be subject to responder or observer bias 45, 46.

## Conclusion

Our study found an inverse association between diarrhoea and reported appropriate treatment of drinking water. We found inverse associations between stunting in children under age two and improved source of drinking water, appropriate drinking water treatment, improved sanitation facility, and complete structure of sanitation facility and a positive association with open defecation. Given the importance of addressing diarrhoea and undernutrition for child survival, implementation research is needed on programs that incorporate water and sanitation interventions and their impact on diarrhoea prevalence and child growth.
